# New Fe_2_O_3_-Clay@C Nanocomposite Anodes for Li-Ion Batteries Obtained by Facile Hydrothermal Processes

**DOI:** 10.3390/nano8100808

**Published:** 2018-10-09

**Authors:** Daniel Alonso-Domínguez, María Pilar Pico, Inmaculada Álvarez-Serrano, María Luisa López

**Affiliations:** 1 Department of Inorganic Chemistry, Faculty of Chemical Sciences, Complutense University of Madrid, Av. Complutense, E-28040 Madrid, Spain; danielad@quim.ucm.es (D.A.-D.); marisal@ucm.es (M.L.L.); 2 Department of R&D, Sepiolsa, Av. Acero, 14-16, Pol. UP-1 (Miralcampo), 19200 Azuqueca de Henares, Guadalajara, Spain; maria.pico@sepiolsa.com

**Keywords:** iron oxide anodes, sepiolite, bentonite, lithium ion battery

## Abstract

New iron-oxide-based anodes are prepared by an environmentally-friendly and low-cost route. The analysis of the composition, structure, and microstructure of the samples reveals the presence of a major hematite phase, which is accompanied by a certain concentration of an oxyhydroxide phase, which can act as a “lithium-reservoir”. By using sodium alginate as a binder, the synthesized anodes display superior electrochemical response, i.e., high specific capacity values and high stability, not only versus Li but also versus a high voltage cathode in a full cell. From these bare materials, clay-supported anodes are further obtained using sepiolite and bentonite natural silicates. The electrochemical performance of such composites is improved, especially for the sepiolite-containing one treated at 400 °C. The thermal treatment at this temperature provides the optimal conditions for a synergic nano-architecture to develop between the clay and the hematite nanoparticles. High capacity values of ~2500 mA h g^−1^ after 30 cycles at 1 A g^−1^ and retentions close to 92% are obtained. Moreover, after 450 cycles at 2 A g^−1^ current rate, this composite electrode displays values as high as ~700 mA h g^−1^. These results are interpreted taking into account the interactions between the iron oxide nanoparticles and the sepiolite surface through hydrogen bonds. The electrochemical performance is not only dependent on the oxidation state and particle morphology, but the composition is revealed as a key feature.

## 1. Introduction

In recent years, the development of new oxide- and carbon-based active materials in the field of energy storage has attracted much attention, especially concerning their potential application in lithium- and sodium-based devices [1,2,3]. In particular, iron-based compounds (e.g., oxides, oxohydroxides) have concentrated the interest of many researchers in the field of lithium ion batteries (LIBs) for their use as anodes in these devices, since they display competitive capacity values [3,4,5]. In addition, these materials are abundant in nature, practically innocuous, and low cost. Thus, compounds as Fe_3_O_4_, γ-Fe_2_O_3_, α-Fe_2_O_3_, and FeOOH present numerous advantages having in mind their high theoretical capacity (~1000 mA h g^−1^) and low environmental cost, i.e., non-toxicity and natural abundance. However, this type of compound shows low stability due to high volume expansion after successive cycles, together with low electrical conductivity, which hinders Li ions insertion-deinsertion processes. In order to surpass these deficiencies, different strategies have been described in recent literature: to obtain nanomembranes [6], to modify the synthesis routes [3], to prepare composites with diverse carbon materials [7], and to optimize the materials response by nano-architecturing them with diverse clays as supports [8]. It is worth mentioning several recent strategies implying the use of carbon fibers obtained from different precursors, leading to a significant improvement of conductivity and structural stability through the discharge-charge processes [9].

Regarding the preparation of bare iron oxide samples, various chemical synthesis techniques have been attempted in order to control their crystal structures and morphologies, such as hydrothermal method, sputtering, electrospinning, electrodeposition, and template method [10,11]. Specifically, hydrothermal synthesis has shown to be propitious over other synthesis techniques in terms of homogeneous nucleation and grain growth of hematite nanocrystals. The experimental parameters such as initial concentrations and temperature reaction time are the main factors determining the structures and morphologies of the obtained α-Fe_2_O_3_ nanocrystals [12]. 

Besides, carbon coating is one of the most effective strategies for improving the structural stability of electrode materials and their cycling performance [13]. For example, PDDA (Polydiallyldimethylammonium chloride, C_8_H_16_ClN)_n_), has been used to fabricate Fe_2_O_3_@C composites starting from FeCl_3_ precursor via a hydrothermal process and further carbonization. These anodes showed improved electrochemical properties: 358 mA h g^−1^ after 150 cycles at 1000 mA g^−1^ current rate (1C), which is three times higher than the value for the bare oxide [14]. In another work, the improvement of the electrochemical performance of the hematite iron oxide was achieved by preparing composites with graphite. This strategy is based on the increase of the conductivity and the minimization of volume changes along the electrochemical cycles. Thus, values of ~300 mA h g^−1^ were reached after 55 cycles at 1000 mA g^−1^ current rate [15]. Graphene has been successfully employed for preparing manganese- and cobalt-doped MFe_2_O_4_@C anodes, reaching specific capacity values of ~900 mA h g^−1^ after 150 cycles at 1000 mA g^−1^ current rate [16]. This system, which has recently been prepared starting from a bimetallic metal-organic framework, displays ~940 mA h g^−1^ at a current rate of 100 mA g^−1^ after 80 cycles [17].

Finally, the study of the electrochemical behavior of different SiO_x_ materials, which present fibrous nature, high specific surface, and low cost, has revealed very promising characteristics [18,19,20]. Among them, sepiolite has been previously used as a template to produce carbonaceous materials [21]. In some cases, the caramelization of sucrose in the adequate conditions permitted to obtain functionalized conducting carbon-sepiolite anodes [22,23]. Further, the preparation of emergent nanocomposites of sepiolite-supported metal oxides led to a significant enhancement of the cycling performance starting from the ~90th cycle [8]. 

Additionally, natural bentonites, mostly employed in heat storage and CO_2_ adsorption applications [24], have been employed in LIBs as a component of a quasi-solid-state electrolyte, which guarantees the battery life for harsh environments [25].

Searching to enhance the cycling performance, the design of a stable electrode capable of minimizing the volume changes is required. Thus, this ideal electrode should contain both a rigid component and internal spaces, permitting in this way large-volume changes without demolishing the structure. Sepiolite is a fibrous clay, which shows both characteristics. We aimed therefore to employ this material as a rigid support of iron oxide nanoparticles in order to improve their electrochemical performance. Complementarily, we searched to further study the possible interesting role of using other clay materials such as bentonite. In this article, we report the preparation and characterization of new iron oxide-carbon-clay nanocomposites, Fe_2_O_3_-Clay@C. We employed a facile and environmentally-friendly hydrothermal route. The natural clays used as supports are sepiolite and bentonite and the binder employed is sodium alginate. Having in mind the above considerations, we have focused our attention in obtaining new nano-architectured anodes through a sustainable process, which could significantly increase the electrochemical performance of these anodes based on bare iron oxides. 

## 2. Materials and Methods

### 2.1. Techniques

X-ray powder diffraction (XRD) patterns were registered at room temperature with a PANanalytical X’PERT POWDER diffractometer (Malvern Panalytical Ltd., Malvern, UK) using Cu (Kα) radiation with λ = 1.5418 Å. The collected data were analyzed by the Rietveld [26] profile method using the Fullprof program [27]. 

Electron microscopy (EM) analyses were made both by scanning (SEM) and high-resolution transmission (HRTEM) techniques. Scanning electron microscopy images were collected with a JEOL JSM 6335F (JEOL, Tokyo, Japan) microscope and HRTEM results were obtained using a JEOL JEM 3000F (300 kV) (JEOL, Tokyo, Japan). Samples were prepared by crushing the powders under n-butanol and dispersing them over copper grids covered with a holey-carbon film. 

Thermogravimetric analyses (TGA) were carried out in a Cahn D200 (ALT, East Lyme, CT, USA) balance at several heating rates under H_2_/He (in 2:3 volume ratio). For the experiments in oxygen atmospheres, thermogravimetric data were obtained by a Pyris thermogravimeter (Perkin Elmer, Waltham, MA, USA), where the analyses were carried out at a heating/cooling rate of 10 °C min^−1^. 

The Fourier transform infrared (FTIR) spectra were recorded on an FTIR Thermo Nicolet 200 spectrophotometer (Thermo Fisher Scientific, Waltham, MA, USA), in the 4000–400 cm^−1^ region at ambient temperature.

The electrochemical tests were performed in Swagelok-type cells assembled in an argon-filled dry box, using Li metal as counter electrode, and a Whatman borosilicate glass fiber sheet (grade GF/D) , saturated with a 1 M LiPF_6_ in ethylene carbonate (EC) and dimethyl carbonate (DMC) (1:1 in weight) as electrolyte. The synthesized samples were mixed with 30% of carbon SP (black carbon from MMM, Willebroek, Belgium) and 10 wt% sodium alginate in deionized water. The resultant slurries were coated onto a copper foil current collector and dried at 80 °C overnight. The resulting electrodes had an active material loading of around 1–3 mg.

Nitrogen adsorption measurements were performed on a Micromeritics ASAP 2010 physisorption analyzer (Micromeritics, Norcross, GA, USA) at 77 K. Brunauer-Emmett-Teller (BET) and Barret-Joyner-Halenda (BJH) methods were used in order to determine the surface area and pore size distribution determination using N_2_ adsorption data. Samples were previously outgassed for at least 4 h at 120 °C.

Solid state ^29^Si NMR-MAS (magic angle spinning nuclear magnetic resonance) spectroscopy was used to characterize the molecular composition of the clays. The experiments were carried out on a Bruker Advance (Bruker, Billerica, MA, USA) 400 MHz spectrometer, with a 9.39 T widebore superconducting magnet, operating at 79.49 MHz, with spin rate of 12 KHz, 90° pulse length of 4.5 µs.

### 2.2. Materials Synthesis and Characterization

The corresponding notations of the synthesized bare iron oxide (FO) and clay-supported samples are included in Table 1. 

The iron oxide-type samples studied in this work were obtained by a hydrothermal process at batch regime and subcritical conditions. For such purpose, iron (II) acetate, Fe(C_2_H_3_O_2_)_2_ (99%, Sigma-Aldrich, St. Louis, MO, USA), solutions with 0.1 M concentration were prepared. These solutions were further introduced in sealed autoclaves and treated at 180 °C for 12 h. These conditions were selected bearing in mind previous experimental research of the authors in similar metal-oxide systems [28]. The obtained precipitates were then filtered through a polymeric membrane (average pore size of about 200 nm; polyethersulphone membranes from Sartorius) and finally dried in a stove at 80 °C (denoted as FO). In order to evaluate the possible role of the precursor solution concentration in the composition of final products, a set of various experiments was carried out in which different molar concentrations of the initial solution were employed, from 0.1 to 0.4 M. A certain amount of an oxyhydroxide-type phase, FeOOH, was always stabilized, which smoothly increased as the initial concentration of the precursor solution increased from 0.1 to 0.4 M, but was not considered as significant (Figure S1). Therefore, the lowest concentration of 0.1 M was selected for further experiments, taking into account cost and ease of handling considerations.

Envisaging the preparation of composites of the FO samples with clays, and taking into consideration the thermal behavior of the employed clays (see below), aliquot portions of the bare iron oxide samples were further heated at selected temperatures 400 and 800 °C (denoted as FO-400 and FO-800, as indicated in Table 1). As expected, the concentration of the FeOOH phase in the samples decreases significantly as temperature increases. Thus, this phase is absent at temperatures near 800 °C. This result was confirmed by FTIR spectra by following the evolution with temperature of the FeOOH typical bands [29], as gathered in Figure S2. 

Clays-hematite composites were prepared by using glucose (Sigma-Aldrich, St. Louis, MO, USA) and the indicated clays. A typical process includes the preparation of 100 mL of a glucose solution 0.35 M, in which the clay is added in a 20% mass percentage. The suspension is then stirred for two hours and further introduced into an autoclave and treated at 180 °C for 6 h. In this way the carbonization of the clays is achieved, and the @C notation is included in the given name of the corresponding samples.

The obtained precipitate is filtered and a mass of 0.2 g is incorporated to 40 mL of the iron acetate 0.1 M solution. The suspension is again treated in an autoclave at 180 °C for 6 h. The obtained precipitate is then filtered and washed with water, ethyleneglycol, and acetone and finally dried in a stove at 80 °C. Portions of these iron oxide-clay@C composites were further heated at 400 and 800 °C and their corresponding notations are indicated in Table 1.

The employed clays were sepiolite and bentonite supplied by Sepiolsa. Their structure and thermal stability were characterized by ^29^Si-MAS-NMR (CP), XRD, and TGA experiments.

Natural mineral sepiolite is a hydrated magnesium silicate fibrous clay of formula Mg_8_Si_12_O_30_(OH)_4_·(H_2_O)_4_·8H_2_O. Its structure is built from continuous tetrahedral (talc-like) and discontinuous octahedral sheets (Figure S3a). Such a framework contains tunnels where zeolitic water molecules are located [30]. Under the adequate thermal treatment, these channels become empty (Figure S3b). Figure S4a shows the ^29^Si-MAS-NMR spectrum of our sepiolite, in which typical signals are appreciated: (i) Q^3^ corresponding to edges of the structural blocks, (ii) Q^3^ ascribed to [SiO_4_] in the inner part of the structural blocks, and (iii) Q^2^ signals related to tetrahedra, in which Si is linked to OH units (silanol groups). 

Besides, bentonite shows montmorillonite-type structure and general formula (Na,Ca)_0.3_(Al,Mg)_2_Si_4_O_10_(OH)_2_·nH_2_O). It is a 2:1 layer mineral, built of sheets of octahedrically coordinated aluminum (^VI^Al in O sheets) which are located each one between two sheets of corner-sharing Si tetrahedra (^IV^Si in T sheets). 

Figure S4b gathers the ^29^Si-MAS-NMR spectrum for our bentonite, in which expected signals are observed: (i) Q^3^ related to Si atoms in the tetrahedral sheets and (ii) Q^3^(1Al)-type signal related to partial substitution of Si^4+^ by Al^3+^ in the tetrahedral sheets of the bentonite structure.

Additionally, Figure S3c shows the TGA curve of the employed sepiolite. The observed steps and the mass losses registered are coherent with that reported in the literature [31] and correspond to the following processes:

(a) up to 200 °C, both hygroscopic and zeolitic water molecules are lost (theoretical mass loss of 11%):
Mg_8_Si_12_O_30_(OH)_4_·(H_2_O)_4_·8H_2_O → Mg_8_Si_12_O_30_(OH)_4_·(H_2_O)_4_ + 8H_2_O
(b) between 250 and 500 °C, the bound water molecules are lost, i.e., those ones which complete the coordination of the terminal Mg^2+^ ions at the edges. For this step, the corresponding theoretical mass loss is 5.7%.
  Let us note that some of the composites prepared by us are heated up to 400 °C. This means that the zeolitic water as well as two of each four bound water molecules are probably lost in them. The partial desorption of the H_2_O species results in the partial collapse of the tunnels [32].
(c) Finally, up to 800 °C, the hydroxyl units are lost and the sepiolite structure is totally decomposed as follows:
Mg_8_Si_12_O_30_(OH)_4_ → 8MgSiO_3_ + 4SiO_2_+ 2H_2_O

## 3. Results and Discussion

### 3.1. Structural and Compositional Characterization

**Figure 1:**
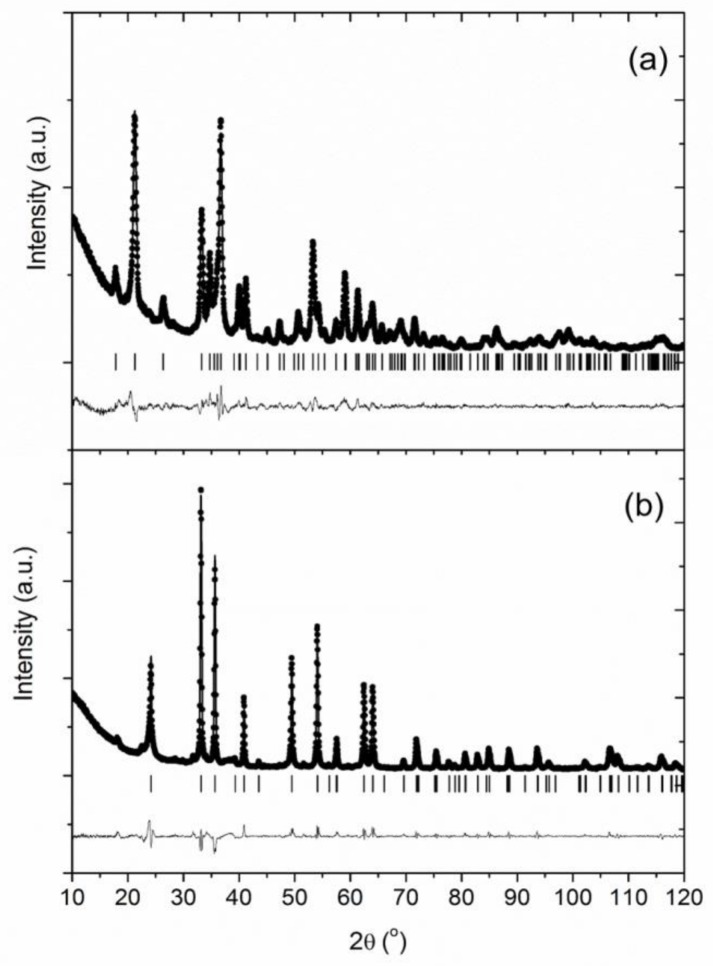
Rietveld refinement profiles from XRD data corresponding to (**a**) intermediate oxyhydroxide phase (α-FeOOH, goethite) and (**b**) Fe_2_O_3_ hematite phase.

Figure 1a,b show the XRD patterns for the prepared FO samples before and after being dried in the oven under 80 °C, respectively. The observed maxima for the undried sample can be indexed as α-FeOOH, goethite (JCPDS 29-713), and Figure 1a shows the corresponding observed, calculated, and difference Rietveld profiles. Otherwise, the XRD pattern of the dried sample (Figure 1b) shows the presence of hematite phase (JCPDS 33-664), which could be accompanied by an additional amorphous hydrated compound. As indicated above (Table 1), this phase is denoted as FO hereafter.

The stabilization of hematite through goethite in processes implying Fe(II) cations has been previously stated [33]. Under certain conditions, the dehydroxilation of lepidocrocite (γ-FeOOH) and heating of goethite, α-FeOOH are known to typically lead to maghemite [34]. However, this ferrihydrite phase can evolve to goethite and further to hematite under soft heating in the presence of Fe(II) cations [35], and it can also be topotactically transformed into hematite [36]. Indeed, it is not unexpected that the dehydration of the intermediate oxyhydroxide leads to hematite by heating at 80 °C (the temperature chosen for the final drying process of our phases). Thus, the use of fresh Fe(II) acetate is critical in our case in order to avoid the presence of magnetite/maghemite phases accompanying the major hematite phase.

Furthermore, the TGA curves under reductive and oxidative atmospheres could be analyzed by considering the presence of hematite phases accompanied by hydrated phases in ~35–45% mass percentages. Representative curves are gathered in Figure S2. The obtained mass changes could be interpreted by considering the following processes:

Under oxygen atmosphere: bounded water is lost in the heating regime, whereas no further mass increase is detected in the subsequent cooling process, coherently with the presence of trivalent iron cations.

Under hydrogen atmosphere: the obtained residues after the TGA experiments could be identified as iron metal. 

Hence, the FO sample is composed of ~55% Fe_2_O_3_ and a ~45% of FeOOH, whereas these mass percentages are ~65 and ~35% in FO-400, respectively. Finally, the FO-800 sample is only formed by hematite. Thus, in our samples the major hematite phase is accompanied by a certain percentage of hydrated oxide phases, probably most consisting in goethite α-FeOOH, which is only absent at high temperatures. The important consequences of this feature on the electrochemical response are commented below.

The XRD patterns for the clay-containing composites were practically identical to those obtained for FO (Figure 1). This is due to the low crystallinity of sepiolite and bentonite compared to the iron oxide phase.

**Figure 2:**
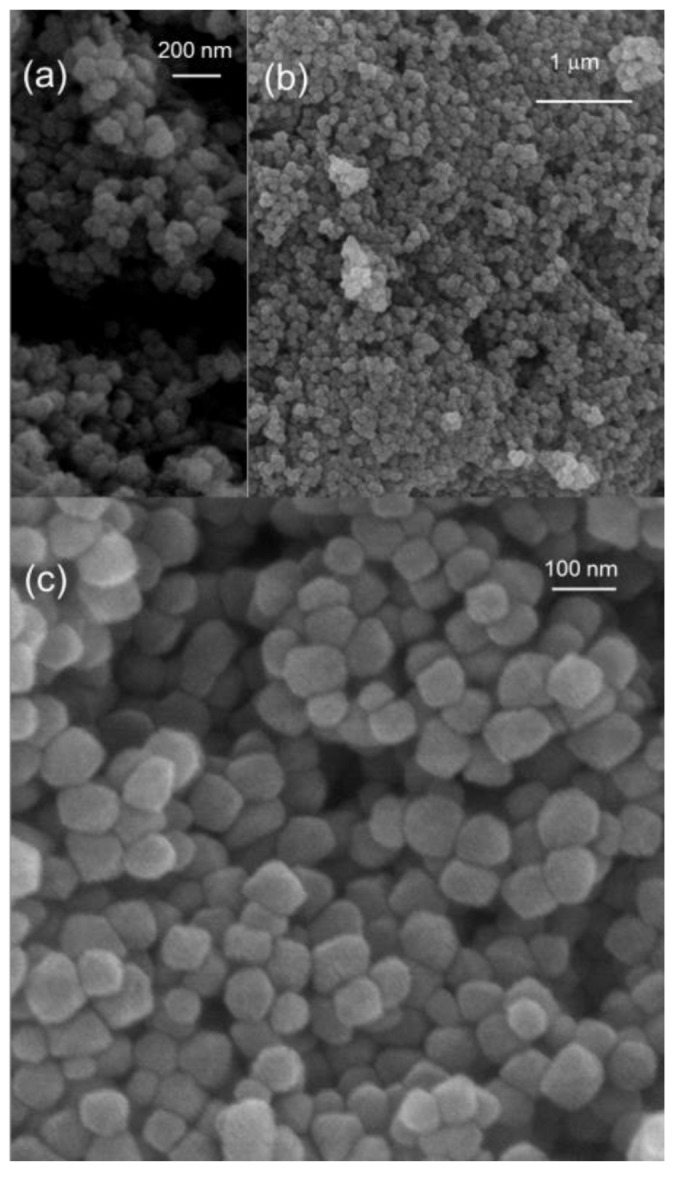
(**a**) SEM images of the obtained iron oxyhydroxide (goethite); (**b**,**c**) SEM images at different magnifications of the obtained iron oxide (hematite), FO sample.

A microstructural analysis of the FO samples by SEM revealed that the goethite precursor phase consists of agglomerated formations with a spongy aspect (Figure 2a), which is coherent with its low crystallinity. Besides, the microstructure of the hematite oxide obtained is composed of pseudo-spheres of about 60 nm with highly monodispersive character, as can be appreciated in Figure 2b,c.

### 3.2. Electrochemical Behaviour

**Figure 3:**
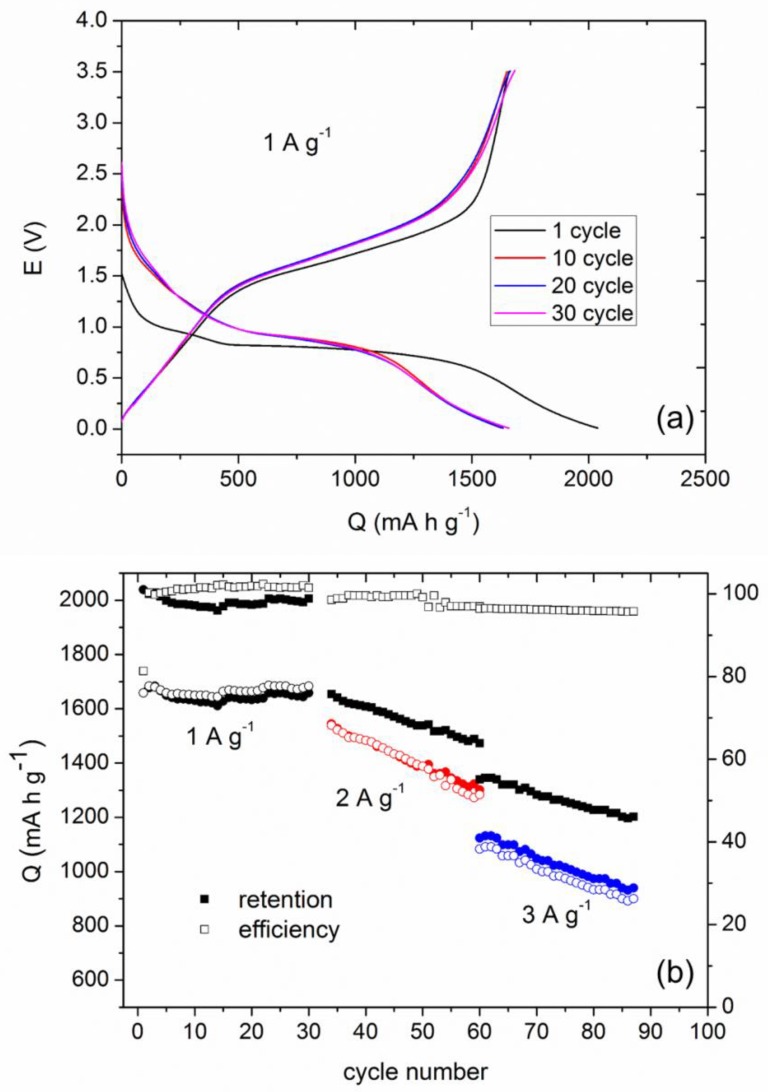
(**a**) Discharge-charge curves at 1C and (**b**) specific capacity versus cycle number at different rates, for the FO composite vs. Li^+^/Li.

The electrode composites were prepared by mixing the active material (the synthesized samples) with carbon and sodium alginate as a binder. Details are given in the experimental section. Figure 3a gathers the galvanostatic discharge-charge curves for the FO electrode at 1C rate for the first cycle and several selected successive cycles. An initial specific capacity value as high as 2040 mA h g^−1^ is obtained, which decreases towards 1600 mA h g^−1^ at the second cycle and is further practically invariant through the subsequent 30 cycles (Figure 3b).

These results are clearly better than those found in hematite anodes obtained in comparable conditions: e.g., hematite nanowires hydrothermally synthesized showed maximum initial charge/discharge capacity of 1303 mA h g^−1^ at the rate of 0.1C, which evolved towards 456 mA h g^−1^ after 100 cycles [37].

**Figure 4:**
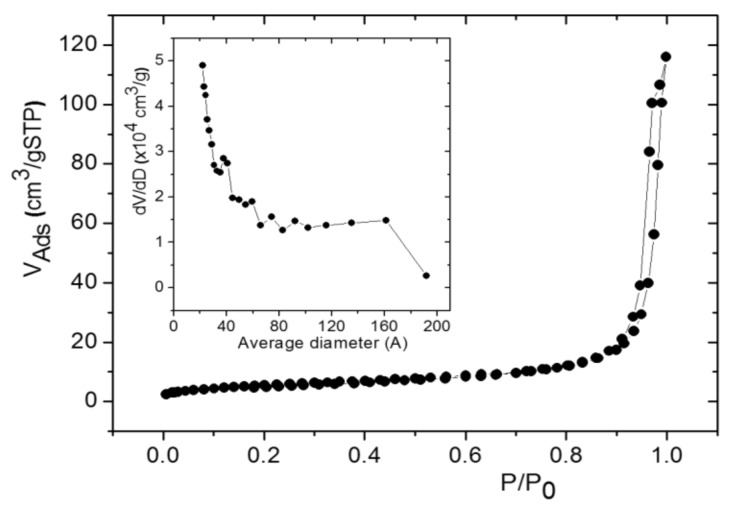
The nitrogen adsorption-desorption isotherm for FO sample. Inset shows the corresponding pore size distribution.

Besides, multiwall carbon nanotubes covered by nanoparticles of hematite displayed an initial discharge capacity of ~2230 mA h g^−1^ at 25 mA g^−1^ current density, which decreased to ~550 mA h g^−1^ at the second cycle and stabilized at ~250 mA h g^−1^ after 30 cycles [38]. Otherwise, iron oxide samples obtained from goethite at 600 °C have been previously reported to present capacity values of ~1100 mA h g^−1^, which decreased to 841 mA h g^−1^ after 40 cycles at 100 mA g^−1^ rate [39]. Their electrochemical response was connected to the porosity of the samples, which showed a BET surface area of 9.6 m^2^ g^−1^. This value is coherent with the expected one for a synthetic hematite obtained at temperatures higher than 500 °C [40]. The BET surface area of the FO samples was measured and higher values of about 19.3 m^2^ g^−1^ were obtained (see Figure 4). These values are closer to those typical of hematite rich soil samples in which the presence of water allows the formation of FeOOH phase. This leads us to confirm the presence of FeOOH in synthetized samples used in this work. The pore size distribution is multimodal though pores with diameters of about 40 Å seem to be predominant.

The FO sample exhibits an initial specific capacity value as high as 2040 mA h g^−1^ (Figure 3a), which, as expected, decreases in the second cycle towards 1600 mA h g^−1^. This effect is commonly related to solid electrolyte interphase (SEI) formation effect, whose importance, composition, and main features have been the focus of numerous recent research papers [41,42,43]. Among the different compounds involved, the presence of LiOH and Li_2_CO_3_ phases has been pointed to be especially leading. Indeed, the preparation of artificial layers by atomic layer deposition has been proposed to lead to enhanced electrochemical properties for carbonaceous anodes [44]. Particularly, LiOH can be formed by in situ hydration of Li_2_O, by water contamination or by simply aging. In this sense, its development could be enhanced if a metal hydroxide-phase is already present in the anode material, through the following process [42]:

FeOOH + 3Li^+^ + 3e^−^ → Fe + Li_2_O + LiOH

**Figure 5:**
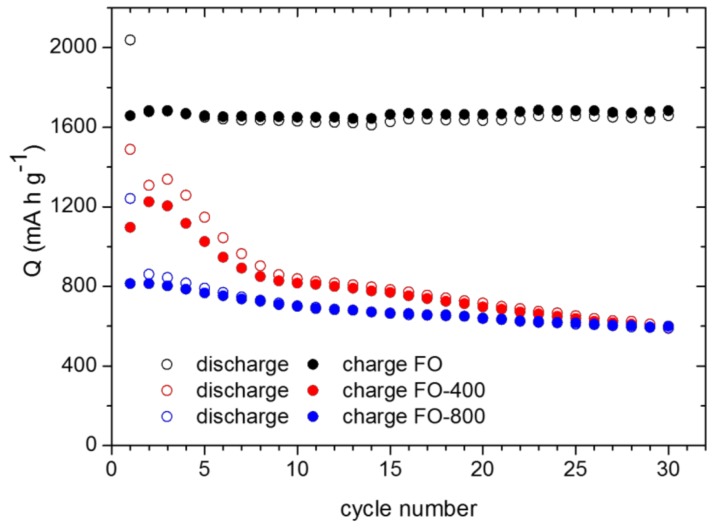
Specific capacity vs cycle number at 1 A g^−1^ for the FO anode.

In Figure 3b, the superior reversibility in the electrochemical response of the FO anodes can be appreciated. Thus, at 1 A g^−1^ current density a specific capacity value of ~1700 mA h g^−1^ is maintained after 30 cycles. The cycling performance is reduced at faster rates, but the capacity value reaches ~900 mA h g^−1^ after 90 cycles even at 3 A g^−1^. This response is better than the previously reported for bare hematite anodes [45] and it is particularly attractive having in mind the easy and innocuous synthesis method employed in our case. Indeed, very promising capacity values have been reported for several iron hydroxides, e.g., 1000 mA h g^−1^ and 1400 mA h g^−1^ for β-FeOOH in microparticles and nanorods form, respectively [5,46]. For α-FeOOH an initial capacity value as high as 1520 mA h g^−1^ has been obtained, which declines rapidly at the first few cycles towards 400 mA h g^−1^ [47]. As stated above, hematite and goethite both coexist in our samples. Thus, as the theoretical capacity values for Fe_2_O_3_ and FeOOH are similar (1007 and 905 mA h g^−1^, respectively), in the absence of emergent phenomena one could expect potential values for the reduction and oxidation processes in our samples roughly independent of their specific composition and of whether they consist in a mixture or not. In this sense, cycling performance of the FO anodes with different ratios of α-Fe_2_O_3_ and α-FeOOH was evaluated. As indicated above, the obtained raw materials, FO, contain an oxyhydroxide phase which progressively disappears under a thermal treatment at 400 and 800 °C. The electrochemical response of the FO-400 and FO-800 anodes was analyzed and Figure 5 shows the corresponding curves, Q vs. cycle number. 

The FO-400 capacity values are lower than FO ones, the lowest values were obtained for the FO-800 sample, in which the goethite phase was absent. Therefore, the possible role of goethite in the SEI formation as a Li^+^ reservoir, via LiOH formation, could be assumed. Mechanisms of such process have indeed been proposed [5]. The pure β-FeOOH actually displays superior lithium storage properties, i.e., a maximum reversible capacity of about 1400 mA h g^−1^, which means an increment of 20–40% with respect to hematite or magnetite compounds. Nevertheless, after cycling at different current rates this value significantly decreases towards ~300 mA h g^−1^ at 0.2 A g^−1^ after 30 cycles. In this sense, the synergic action of the Fe_2_O_3_-FeOOH mixture present in our FO samples seems to be the key role ruling their outstanding performance. Interestingly, the important role of oxyhydroxide-oxide phases mixture concerning the morphology and the final response of the materials has been pointed out [48].

On the other hand, in order to evaluate the practical extension of the response of a given electrode, it is very interesting to incorporate it as a full cell-component and to characterize the resulting electrochemical behavior [49]. Thus, the behavior of our FO sample versus a conventional cathode was evaluated. The LiNi_0.5_Mn_1.5_O_4_ cathode, LNM hereafter, was chosen and prepared by us in microspheres form. Details of the synthesis and characterization can be found elsewhere [50]. Thus, a FO//electrolyte (1 M LiPF_6_ EC/DEC 1/1)//LNM full cell was assembled and tested in the 2–4.5 V potential window. This cell was prepared with fresh LNM cathode and the pre-cycled anode (two cycles at 0.5 A g^−1^). The FO/LNM ratio of anode: cathode active masses was 0.17. Therefore, in this cell the cathode is defective and limits the cell capacity (the theoretical value of this ratio is 0.14). 

**Figure 6:**
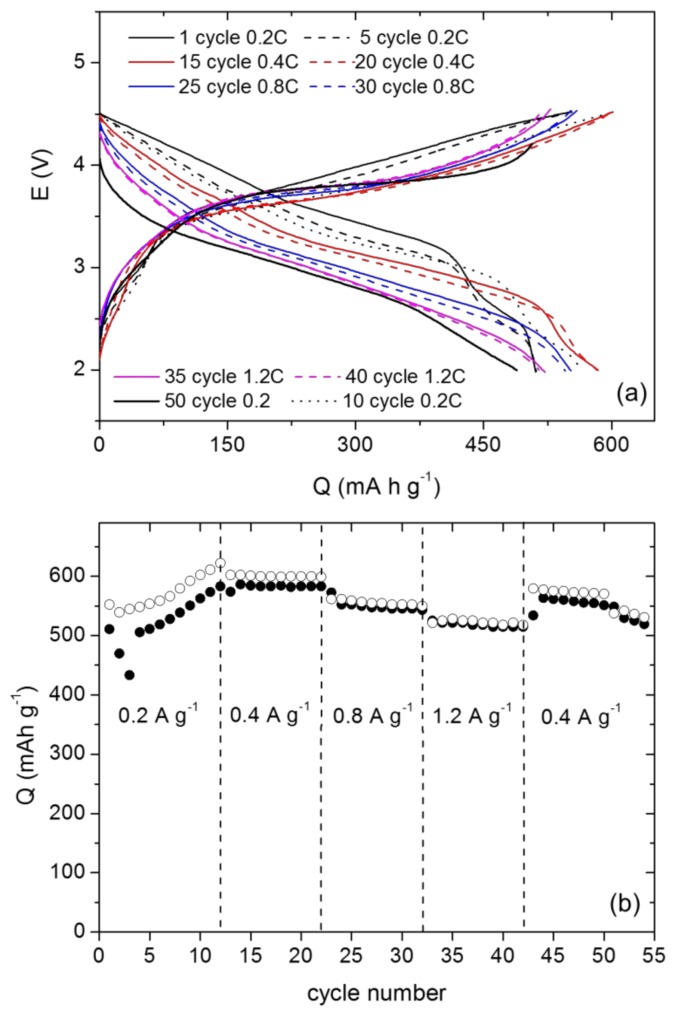
(**a**) Discharge-charge curves and (**b**) rate capability of the FO/LNM full cell at various current densities (LNM refers to LiNi_0.5_Mn_1.5_O_4_ cathode).

Figure 6 gathers the corresponding voltages profiles and Q vs. cycle number at different current rates. The average discharge voltage of the cell was close to 3 V (Figure 6a) and this value agrees with the theoretical one taking into account the partial lithiation of the negative electrode. However, this average discharge voltage changes with the rate and cycle number. This variation in the voltage with the current density is probably a consequence of a polarization increase influenced by the current density typical of conversion anodes, as occurs in similar systems [50,51]. The FO/LNM cell displays an initial capacity increase upon the first 12 cycles at 0.2 A g^−1^ (Figure 6b). This fact indicates the activation of the LNM cathode when assembled with the FO anode. One of the most noteworthy aspects is the influence of the current density. Thus, this cell was cycled at 0.2, 0.4, 0.8, and 1.2 A g^−1^ rates and, even though the discharge capacity decreases as the density current increases, the retention after 42 cycles was as high as 90%. Furthermore, when the current density was again reduced to 0.4 A g^−1^, the specific capacity reached a value near to 600 mA h g^−1^. It can be a retention of 96% after 50 cycles. These results point out a good electrochemical performance for this cell. 

Finally, the efficiencies obtained to all cycles were between 100 and 96%, evidencing a good reversibility. Thus, the FO composite material displays very promising features as an anode in electrochemical devices.

**Figure 7:**
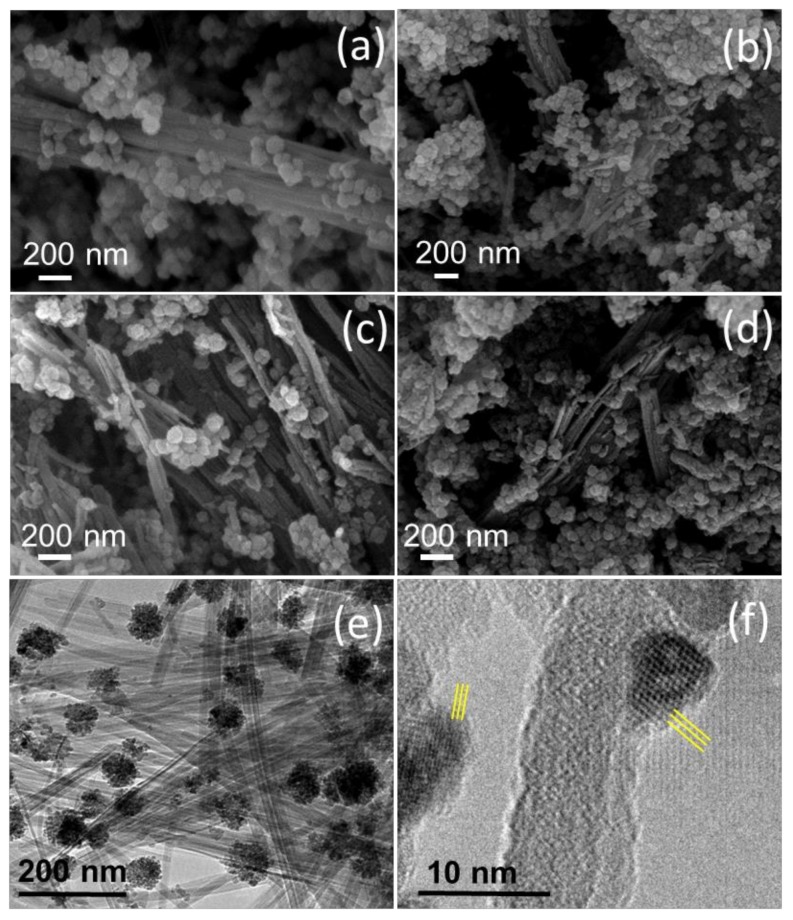
SEM images for (**a**) FO-Sep@C; (**b**) FO-Ben@C; (**c**) FO-Sep@C-400; and (**d**) FO-Ben@C-400; (**e**,**f**) HRTEM images for FO-Sep@C at different magnifications. Yellow lines indicate interplanar distances.

As commented above, we have been interested in exploring the potential improvement of the FO anodes behavior when prepared as nanocomposites with two different clays: sepiolite and bentonite. Thus, two samples were prepared from iron (II) acetate solutions by adding selected masses of glucose, sepiolite, and bentonite, as detailed in the experimental section. Figure 7 shows typical EM images for both nanocomposites, before and after thermal treatment at 400 °C, denoted as FO-Sep@C, FO-Bent@C, FO-Sep@C-400, and FO-Ben@C-400, respectively. In Figure 7, it can be appreciated that the clays serve as a rigid support of the hematite nanospheres, optimizing in this way their available surface for lithium ion interchange. The nanospheres of about φ~60 nm, previously observed by SEM (Figure 2 and Figure 7a–d), consist of aggregated unities of φ~5–10 nm, as revealed by HRTEM images (Figure 7e,f). The presence of the clay facilitates the disaggregation of such nanospheres and the dispersion of the unities, which become anchored on the surface (Figure 7f). Interplanar distances can be appreciated in the nanoparticles, evincing in this way their crystalline nature. Significant differences are not observed for the composites treated at 400 °C (Figure 7c,d).

**Figure 8:**
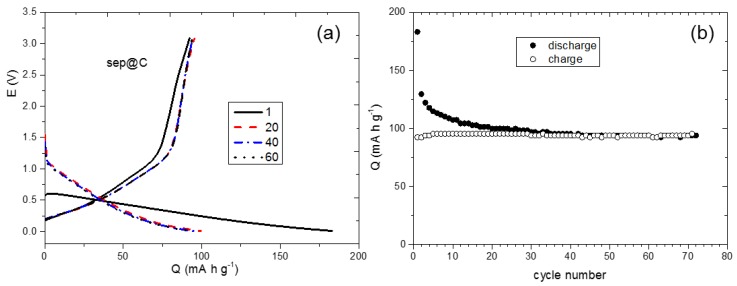
(**a**) Discharge-charge curves and (**b**) rate capability of Sep@C at various current densities.

In order to estimate the contribution of the bare clay to the electrochemical response of the composites, the cycling performance of Sep@C was evaluated at 0.5 mA g^−1^ in the cutoff voltage range of 0.001–3.0 V (Figure 8). The specific capacity at the end of the 70th cycle is stable and corresponds to 100 mA h g^−1^, similarly to previous results obtained for other composites with carbon-sepiolite [52]. These authors reported high values of specific capacity close to 600 mA h g^−1^ at C/20, which dropped down quickly as the density current increased. In our case, sepiolite represents only a 10% of the composite mass and the experiments were made at high current density. For this reason, Sep@C is not an active material for Li storage and does not contribute an evident reversible capacity by itself.

**Figure 9:**
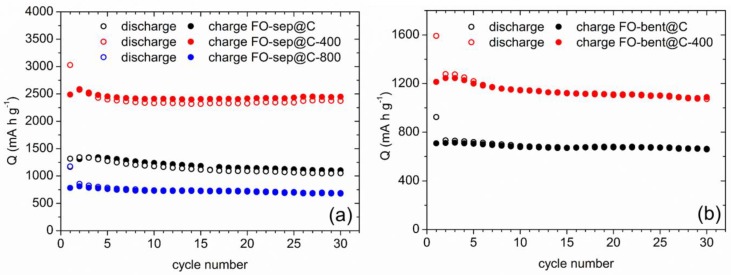
Specific capacity vs. cycle number for different samples of (**a**) FO-Sep@C and (**b**) FO-Ben@C.

In Figure S5, the charge-discharge curves of FO-Sep@C and FO-Ben@C are shown. Very good electrochemical response is obtained in the case of the sepiolite composites, probably due to the higher structural stereoaffinity between this clay and the iron oxide [53]. Nevertheless, the capacity values for the clay-containing composites are smaller than in the case of the pristine anode FO. Thus, after 30 cycles at 1 A g^−1^, FO-Sep@C and FO-Ben@C deliver ~1300 and ~650 mA h g^−1^, respectively, whereas the FO electrode exhibits ~1600 mA h g^−1^. Both samples were heated at 400 °C in order to evaluate the influence of the FeOOH compound in their electrochemical response. The corresponding samples, FO-Sep@C and FO-Ben@C, were tested and the capacity curves vs. cycle number are shown in Figure 9 with comparing purpose.

**Figure 10:**
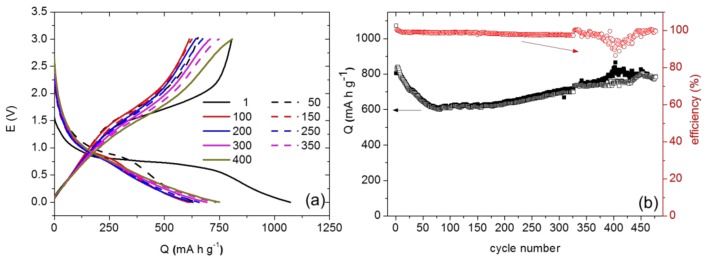
(**a**) Discharge-charge curves at 2C and (**b**) specific capacity versus cycle number at different rates, for the FO-Sep@C-400 composite vs. Li^+^/Li.

All the composites show very good cycling stability. Thus, the obtained retentions in discharge process after 30 cycles (taking into account the second cycle in each case) are about 78, 92, 91, and 84% for FO-Sep@C, FO-Sep@C-400, FO-Ben@C, and FO-Ben@C-400, respectively. Interestingly, the sample FO-Sep@C-400 displays an extraordinary performance with an initial capacity value of ~3000 mA h g^−1^, which smoothly decreases after the cycling process leading to a retention greater than 90% (from the 2nd cycle) and displaying capacity values of ~2500 mA h g^−1^ after 30 cycles at 1 A g^−1^. It is worth underlining that to our knowledge such a high initial discharge capacity for hematite-clay nanocomposites has not been previously reported. Moreover, the FO-Sep@C-400 electrode shows a superior cycling stability. Indeed, specific capacity values of ~770 mA h g^−1^ after 450 cycles are obtained when this sample is cycled at 2C (Figure 10). 

**Figure 11:**
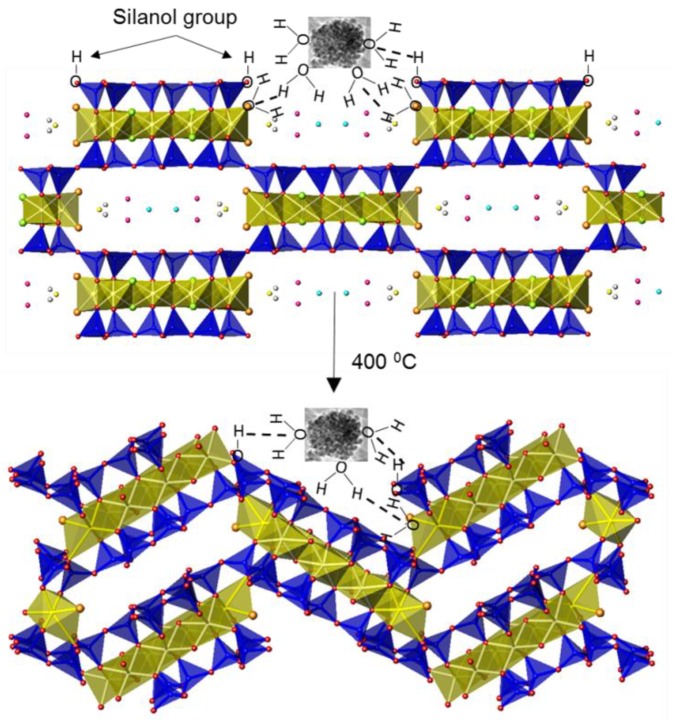
Schematic representation of the interaction between iron oxide nanoparticles and sepiolite surface via hydrogen bonds, in the FO-Sep@C-400 electrode.

Aiming to understand this outstanding response, the actual compositional features of this sample were analyzed. As indicated above, at 400 °C the zeolitic water and a half of the bounded water molecules are lost and the resulting structure is somewhat collapsed (see Figure S4). These changes seem to permit a better anchoring of the iron oxide phase over the sepiolite fibers, giving rise to anode materials with better electrochemical performance. Hence, the clays act as a rigid support, which facilitate a reduction of the volume stress linked to the cycling process. As it has already been suggested by other authors [54], the interaction between iron oxide and sepiolite is due to a solvation layer. In this way, even at 400 °C the iron oxide nanoparticles are solvated while at 800 °C both sepiolite and iron oxide particles are completely water-free (see Figures S1 and S2 and corresponding FTIR bands assignment). Thus, in the FO-Sep@C-400 anode the sepiolite surface and the iron oxide nanoparticles probably interact through hydrogen bonds, as previously stated for similar systems [54]. Figure 11 gathers a schematic representation of such interaction.

This could indicate that the dispersion of the particles into the sepiolite matrix results from solvation of silanol groups or coordinated water. Thus, in the FO-Sep@C-400 sample the sepiolite fibers are weaved in such a way that the iron nanoparticles are held over them. This interaction permits to optimize the contact between both composite components and leads to their excellent electrochemical behavior.

In summary, comparing the electrochemical responses of FO-Sep@C and FO-Ben@C materials, their electrochemical response is optimal when zeolitic water molecules are eliminated by means of a thermal treatment at 400 °C. Indeed, when the sample is further heated at 800 °C, and the clay structure is totally destroyed, the capacity values are significantly decreased (Figure 9a, FO-Sep@C-800 sample). Besides, sepiolite is revealed as a better rigid support for hematite nanoparticles, probably due to stereoaffinity considerations.

## 4. Conclusions

Environmentally-friendly and low-cost, new iron-oxide anodes were prepared displaying superior electrochemical response with high specific capacity values and high stability. 

The iron-oxide anodes contain an oxyhydroxide phase, which is revealed as a key component regarding the delivery of this excellent behavior, not only versus Li but also versus a high voltage cathode in a full cell. This response is further optimized for the clay-supported composites, particularly for sepiolite-containing electrodes. The composites treated at 400 °C show the best response. At this temperature, the clay surface becomes optimal for the adequate support of the iron oxide nanoparticles. High capacity values of ~2500 mA h g^−1^ after 30 cycles at 1 A g^−1^ and retentions close to 92% are obtained. Further, the FO-Sep@C-400 composite displays an outstanding cycling stability: specific capacity values of ~770 mA h g^−1^ after 450 cycles when cycled at 2 A g^−1^. To our knowledge, such high values have been previously reported only for Si anodes. This response can be interpreted in terms of an optimal interaction between iron oxide nanoparticles and sepiolite surface through hydrogen bonds, related to the particular structural and compositional features of this sample. Therefore, the hydrothermal method employed in this work has permitted to successfully prepare nanoarchitectured anodes which display outstanding performance.

## Figures and Tables

**Table 1:** Notation of the samples.

Notation	Clay	T(°C) ^1^
FO	-	-
FO-400	-	400
FO-800	-	800
Sep@C	sepiolite	-
FO-Sep@C	sepiolite	-
FO-Sep@C-400	sepiolite	400
FO-Sep@C-800	sepiolite	800
FO-Ben@C	bentonite	-
FO-Ben@C-400	bentonite	400

^1^ Temperature of further heating from hydrothermal processes. FO stands for Fe_2_O_3_, Sep for sepiolite, Bent for Bentonite and @C for Carbon.

## Supplementary Materials

The following are available online at http://www.mdpi.com/2079-4991/8/10/808/s1, Figure S1: TGA curves (a) for FO samples under oxygen atmosphere; (b) under H_2_:He (3:1) atmosphere for FO samples prepared from precursor solutions of different concentration and (c)under H_2_:He (3:1) atmosphere FO samples obtained at different temperatures, Figure S2: FTIR spectra of FO and FO-800 °C, Figure S3: Schemes of sepiolite structure (a) before and (b) after (dehydrated) thermal treatment; (c) TGA and DTA curves of sepiolite, Figure S4: ^29^Si-MAS-RMN spectra and bands assignments for the employed (a) sepiolite and (b) bentonite, Figure S5: Discharge-charge curves of of FO-Sep@C, FO-Ben@C, FO-Sep@C-400, FO-Ben@C-400 and of FO-Sep@C-800.
