# Supplementary material for: New Fe2O3-Clay@C Nanocomposite Anodes for Li-Ion Batteries Obtained by Facile Hydrothermal Processes
Source: Nanomaterials (Basel). 2018 Oct 9;8(10):808. doi: 10.3390/nano8100808 (PMC6215114; doi:10.3390/nano8100808)
Supplement: Supplementary file 1 [file nanomaterials-08-00808-s001.pdf]

Supporting Information

New Fe_2O_3-Clay@C nanocomposite anodes for Li-ion batteries obtained by facile hydrothermal processes.

Daniel Alonso-Domínguez[1], María Pilar Pico [2], Inmaculada Álvarez-Serrano[1,*] and María Luisa López[1]

[1] Dpto. Química Inorgánica I, Facultad de Ciencias Químicas, Universidad Complutense de Madrid, avda. Complutense s/n, E-28040 Madrid, Spain, danielad@quim.ucm.es, marisal@ucm.es

[2] Sepiolsa, Avda. del Acero, 14-16, Pol. UP-1 (Miralcampo), 19200-Azuqueca de Henares, Guadalajara, Spain; maria.pico@sepiolsa.com

* Correspondence: ias@ucm.es; Tel.: +34-91-394-5237

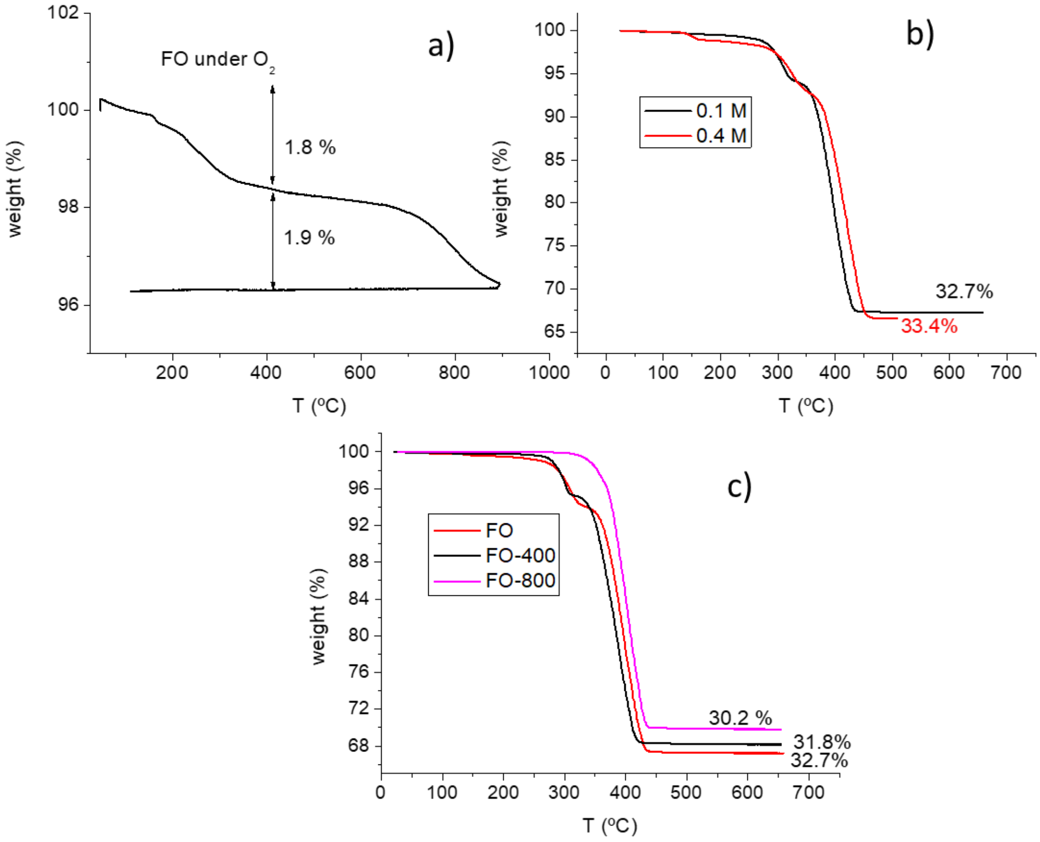

Figure S1 TGA curves a) for FO samples under oxygen atmosphere; b) under H_2:He (3:1) atmosphere for FO samples prepared from precursor solutions of different concentration and c)under H_2:He (3:1) atmosphere FO samples obtained at different temperatures.

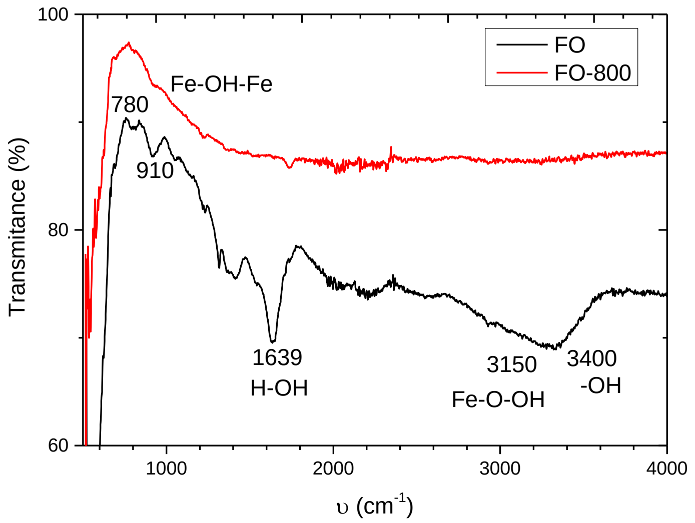

Figure S2 FTIR spectra of FO and FO-800ºC

The two peaks at 1639 and 3400 cm^{-1} can be assigned to the vibration bending and stretching modes of O-H, indicating the presence of adsorbed water on the surface of the sample. The peaks at 780 and 910 cm^{-1} can be related to the in-plane bending of surface hydroxyl groups, i.e. Fe-OH-Fe. In addition, the band at 3150 cm^{-1} is related to the stretching mode of Fe-O-OH in α-FeOOH. These bands disappear after the thermal treatment at 800ºC. This is coherent with the TGA curve under oxygen atmosphere for the prepared iron oxide samples, which indicates that the FeOOH phase totally decomposes into hematite at that temperature (Figure S1a).

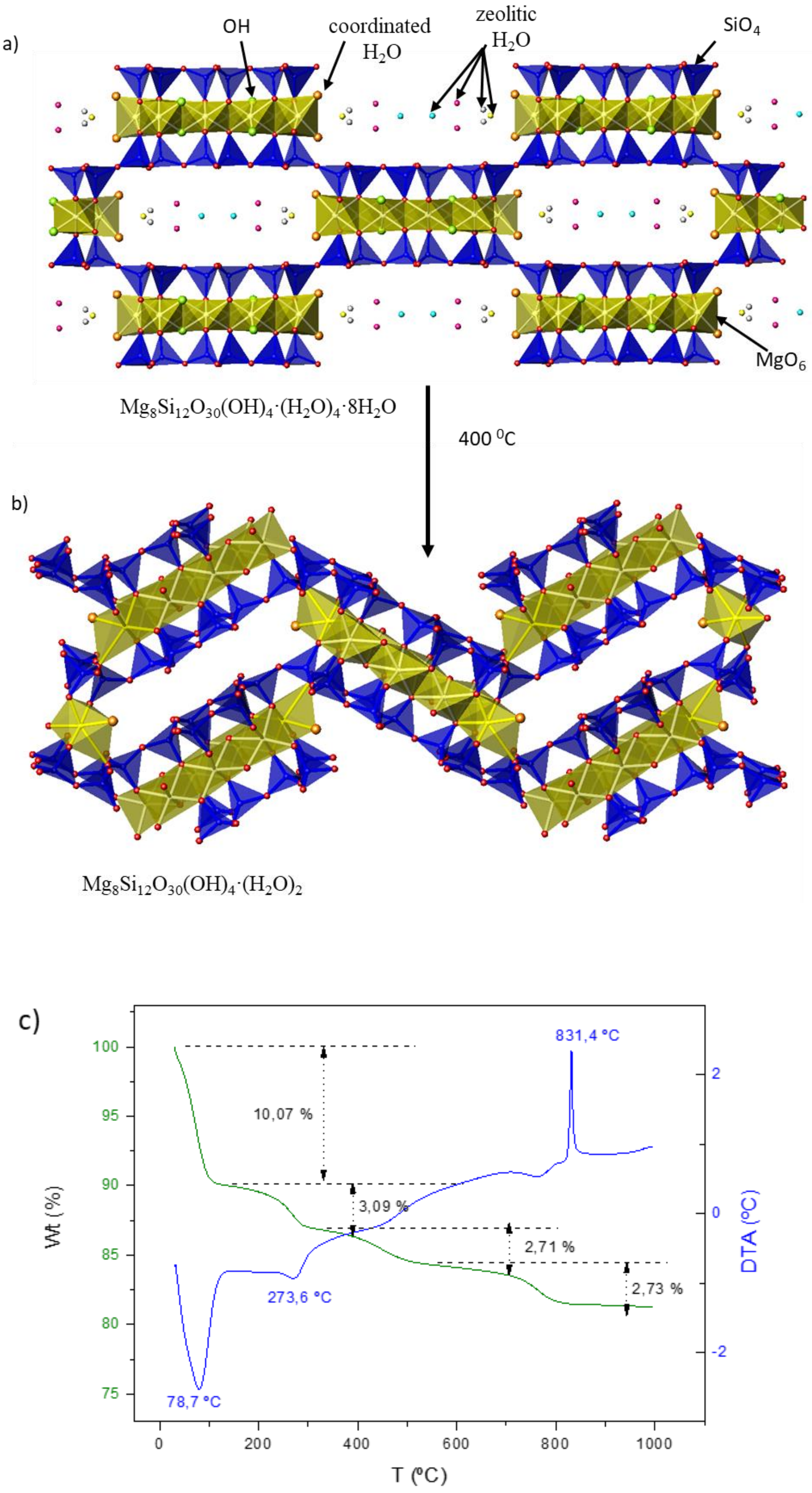

Figure S3 Schemes of sepiolite structure a) before and b) after (dehydrated) thermal treatment; c) TGA and DTA curves of sepiolite.

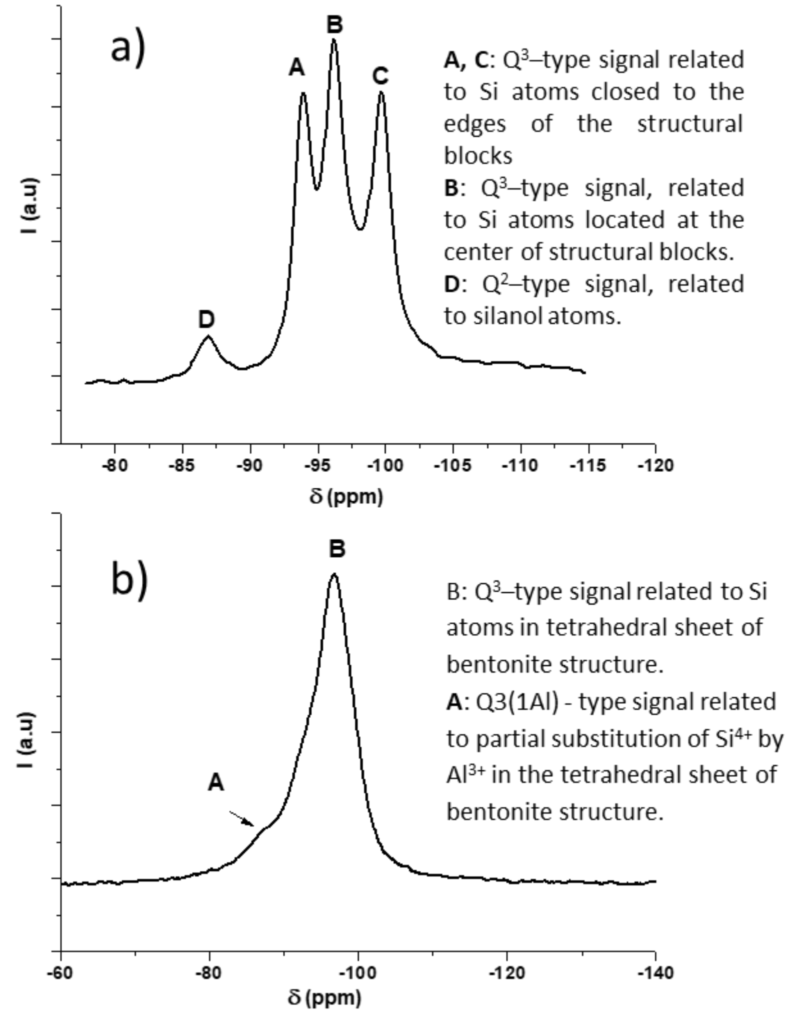

Figure S4 ^{29}Si-MAS-RMN spectra and bands assignments* for the employed a) sepiolite and b) bentonite

*For deeper insight about assignments see e.g. the following references:

-J.L. Valentín, M.A. López-Manchado, P. Posadas, A. Rodríguez, A. Marcos-Fernández, L. Ibarra, *J.Colloid Interface Sci.* **298** (2006) 794–804

- E. Pomakhina, D. Deneele, A. Gaillot, M. Paris, G. Ouvrard, *Cement and Concrete Research,* **42** (2012) 626–632.

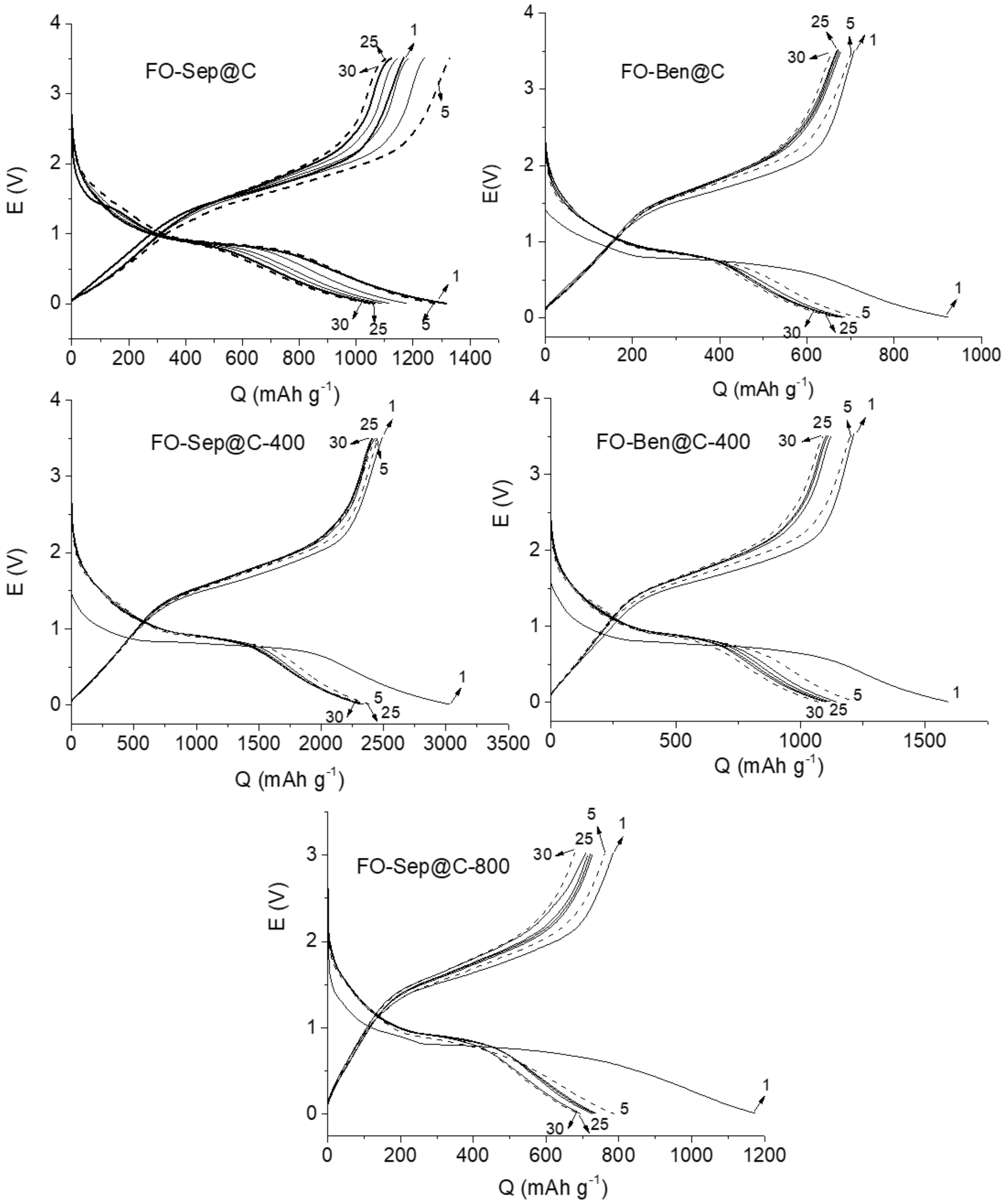

Figure S5 Discharge-charge curves of of FO-Sep@C, FO-Ben@C, FO-Sep@C-400, FO-Ben@C-400 and of FO-Sep@C-800.
